# The impact of helminth-induced immunity on infection with bacteria or viruses

**DOI:** 10.1186/s13567-023-01216-3

**Published:** 2023-10-03

**Authors:** Hong Chen, Zengguo Cao, Mingyuan Liu, Michael S. Diamond, Xuemin Jin

**Affiliations:** 1 State Key Laboratory for Zoonotic Diseases, Key Laboratory for Zoonosis Research, Ministry of Education, Institute of Zoonosis, College of Veterinary Medicine, Jilin University, Changchun, China; 2 State Key Laboratory of Virology, Key Laboratory of Special Pathogens and Biosafety, Wuhan Institute of Virology, Chinese Academy of Sciences, Wuhan, 430071 China; 3 Jiangsu Co-innovation Center for Prevention and Control of Important Animal Infectious Diseases and Zoonoses, Yangzhou, Jiangsu China; 4 Departments of Medicine, Molecular Microbiology, Pathology, and Immunology, Washington University School of Medicine, St Louis, MO USA

**Keywords:** Helminth, viruses and bacteria, co-infection, type 2 immune response, SARS-CoV-2

## Abstract

Different human and animal pathogens trigger distinct immune responses in their hosts. The infection of bacteria or viruses can trigger type I pro-inflammatory immune responses (e.g., IFN-γ, TNF-α, T_H_1 cells), whereas infection by helminths typically elicits a type II host resistance and tolerizing immune response (e.g., IL-4, IL-5, IL-13, T_H_2 cells). In some respects, the type I and II immune responses induced by these different classes of pathogens are antagonistic. Indeed, recent studies indicate that infection by helminths differentially shapes the response and outcome of subsequent infection by viruses and bacteria. In this review, we summarize the current knowledge on how helminth infections influence concurrent or subsequent microbial infections and also discuss the implications for helminth-mediated immunity on the outcome of SARS-CoV-2 disease.

## Introduction

Many viruses and bacteria are threats to human and animal health. In the laboratory, the pathogenesis of viral and bacterial infections is often studied in model organisms under specific pathogen-free conditions [1]. However, in nature, co-infections with viruses, bacteria, and helminths are the norm, and infection with one organism can alter host susceptibility to infection with another [2, 3]. Helminths have coevolved with their vertebrate hosts for hundreds of millions of years, which has enabled many to persist chronically with limited tissue damage [1]. Moreover, their hosts have developed tolerance mechanisms as a strategy to prevent the adverse effects of helminth-mediated or immune-mediated tissue damage.

Helminths including nematodes, cestodes and trematodes, are handled differently by the host immune system compared to bacteria or viruses. Bacteria and viruses both typically trigger a type 1 immune response [4]. Although the development of such pro-inflammatory responses is crucial for the control of potentially lethal bacteria and virus infections, the cost can be tissue damaging inflammation [3]. In contrast, helminths stimulate potent type 2 immune response, which results in clearance and/or tolerance to helminths, and includes anti-inflammatory and wound healing programs. These properties are important when large multicellular helminths migrate through host tissues [5]. Recent reviews explored some recent literature to understand the main immune mechanisms on the control of viral coinfection [6, 7]. In the context of co-infections, published studies suggest that helminth infection can either be beneficial or detrimental to bacterial and viral infection [8, 9]. Despite recognizing these different outcomes, there is limited mechanistic insight as to the basis for these effects. Rather than an exhaustive summary of the literature, our goal in this review is to provide some key concepts and emphasize existing issues in relation to helminth as a friend or foe in bacterial or viral infection.

## Type 2 immune response generated by helminth infection

Parasitic helminths typically establish chronic infection, yet are generally tolerated with limited immunopathology, presumably due to their potent immunomodulatory effects [10]. Helminth infections generally induce robust type 2 immune responses [11], with protective immunity mediated by T_H_2 cells and the cytokines they produce, including IL-4, IL-5, IL-9, and IL-13 [12]. While T_H_2 responses limit helminth infection and can result in the physical expulsion from the mucosal membranes in which they reside, helminths are rarely killed. The importance of the T_H_2 response in helminth immunity is supported by population genetic associations between loci that control T_H_2 responses or their effector cytokines and susceptibility to worm infections [13]. Moreover, helminths were not expelled from the mouse intestine in the absence of IL-4/IL-13, the IL-4Rα chain (a subunit of the IL-4 and IL-13 receptors), or STAT6 (a molecule that mediates IL-4 and IL-13 signal transduction) [14–16]. A deficiency of IL-5 or IL-9 in mice also resulted in much greater worm burden during acute or chronic infection [17, 18].

The epithelial cell barrier is often the first line of defense against helminths, but also provides signals that instruct dendritic cells (DCs), group 2 innate lymphoid cells (ILC2s), and T_H_2 cells to produce type 2 immune responses [19]. In response to helminths or ensuing mast cells responses, intestinal epithelial cells (IECs) release IL-33, which binds to its receptor, ST2 (suppression of tumorigenicity 2), to activate a wide range of immune cells and elicit the production of type 2 cytokines by ILC2s, T_H_2 cells, basophils, and mast cells. Tuft-cell-derived IL-25 is necessary for activating IL-13 production by ILC2s, further promoting tuft cell expansion and providing an early positive-feedback loop that amplifies the type-2-cell-mediated response [20]. Thymic stromal lymphopoietin (TSLP) is also produced by epithelial cells, which promotes T_H_2 cell differentiation and cytokine production and can act on a wide array of immune cells [21]. Mucus production from goblet cells is induced by type 2 cytokines, with IL-13 and IL-4 together playing dominant roles [22, 23]. ILC2s express IL-5 and IL-13, and IL-4, under certain circumstances [24, 25]. IL-4 helps to mediate antibody class switching and the production of IgE. IgE along with antigen forms immune complexes that bind to IgE receptors on basophils and mast cells, resulting in allergic responses and the release of vasoactive and gastrointestinal (GI) tract motility mediators including histamine and serotonin [26]. IL-5 is responsible for the activation and recruitment of eosinophils from the bone marrow into sites of inflammation [27]. IL-9 acts as a growth factor of mast cells, promoting the proliferation and survival of mast cells [28]. IL-13 induces smooth muscle movement, goblet cell hyperplasia, subepithelial fibrosis, and mucus hypersecretion [29].

## The co-infection niche

Most helminths enter their hosts via the fecal-oral route in the form of embryonated eggs or infective larvae usually through consumption of contaminated water or food. Some of the GI tract helminths undergo developmental molts to generate mature adult larvae that establish infection in the GI tract (e.g., *Heligmosomoides polygyrus*), whereas others cross the intestinal mucosal barrier to the circulatory system and invade skeletal muscle tissues (e.g., *Trichinella spiralis*). Others enter the body via skin penetration in the form of infective larvae (e.g., *Nippostrongylus brasiliensis*). Although the parasitic locations of different helminths vary, the adaptation of worms to their mammalian hosts and their particular immune evasion strategies enable them to survive with limited tissue damage [30].

The effects of acute or chronic helminth infection can be beneficial or detrimental to subsequent bacterial or viral infection depending on the organism and location of infection. Enteric helminth *H. polygyrus* enhanced susceptibility in the intestine of mice to some enteric pathogens such as *Citrobacter rodentium* [31], *Salmonella typhimurium* [32], and West Nile virus (WNV) [33]. In comparison, in the lungs, *H. polygyrus* had protective antiviral effects in the context of respiratory syncytial [34] or influenza [35] virus infection. In addition, worm infection can have effects on remote sites. For instance, an acute helminth infection (*N. brasiliensis*) induced a type 2 immune profile in the female genital tract, which leads to greater epithelial ulceration and pathology in the context of subsequent herpes simplex virus (HSV)-2 infection [36]. *T. spiralis*, which inhabits the small intestine for approximately 2 to 3 weeks, facilitated greater intestinal tissue infection of an enteric norovirus in mice [37] yet ameliorated influenza virus-induced inflammation in the lungs [38] and *Pseudomonas aeruginosa*-induced pneumonia. The helminth *Schistosoma spp.*, which can invade visceral tissues including lungs and the liver, also protected lungs from infection with influenza virus or pneumonia virus in mice [39]. Thus, helminths can colonize different niches, and the influence on other microbial agents appears to differ depending on the site of secondary infection, with a general protective effect on infection and disease caused by respiratory bacteria or viruses [40].

## Bacteria and helminth co-infections

*S. typhimurium* is used as a model for human typhoid fever and its deleterious effects in mice have been shown to be modulated by helminth infection [41]. Levels of colonization of *S. typhimurium* increased independently of regulatory T or T_H_2 cells induced by co-infection with *H. polygyrus*. Instead, small intestinal metabolites, which are altered in abundance during helminth infection, promoted expression of *Salmonella* pathogenicity island 1 (SPI-1) genes and increased intracellular invasion [42]. It remains unclear, which helminth-induced metabolite is responsible for the worsened *Salmonella* infection. Consistent with these results, anti-helminthic treatment prior to *Salmonella* challenge restored host resistance to *Salmonella* [32]. These data suggest that the presence of helminths supports initial *Salmonella* colonization in the host small intestine.

Mycobacterial interactions with helminth infections have also been studied. In one experimental model, infection of mice with *Schistosoma mansoni* made the animals more susceptible to *Mycobacterium bovis* (BCG) infection. The induction of dominant T_H_2 type responses by helminth infection resulted in an impaired T_H_1 type response to BCG [43]. Others have reported that mice infected with the intestinal helminth *N. brasiliensis* have impaired resistance to airborne *M. tuberculosis* infection and accumulate higher bacterial burden in the lungs of coinfected mice [44]. In this case, the T_H_2 response induced by *N. brasiliensis* did not impair *Mtb*-specific T_H_1 cellular immune responses, but instead enhanced the intracellular persistence of *M. tuberculosis*, in part by inducing alternatively activated macrophages via an IL-4Rα signaling pathway [44]. Experiments with *S. mansoni* co-infection or immunization with *S. mansoni* egg antigens showed impaired *M. tuberculosis*-specific T cell responses without affecting macrophage-mediated *M. tuberculosis* control [45]. *S. mansoni* infection resulted in an accumulation of high arginase-1–expressing macrophages in the lung, which formed type 2 granulomas and exacerbated inflammation in *Mtb*-infected mice. Treatment of coinfected animals with an anti-helminthic drug improved *Mtb*-specific T_H_1 responses and reduced disease severity [45].

Similar results have been observed in co-infected mice with *C. rodentium*, an extracellular mouse-specific enteric pathogen used to model pathogenic *Escherichia coli* infections and inflammatory bowel disease [46]. Mice coinfected with *H. polygyrus* and *C. rodentium* developed substantial pathology in the colon that was associated with increased bacterial burden, morbidity, and mortality; this enhanced disease required STAT6-mediated type 2 immune mechanisms [31]. *H. polygyrus* and *C. rodentium* co-infected MyD88 knockout mice accumulated higher levels of T_H_2 cytokines during helminth infection [47], and sustained greater mortality than wild-type mice [48].

**Figure 1:**
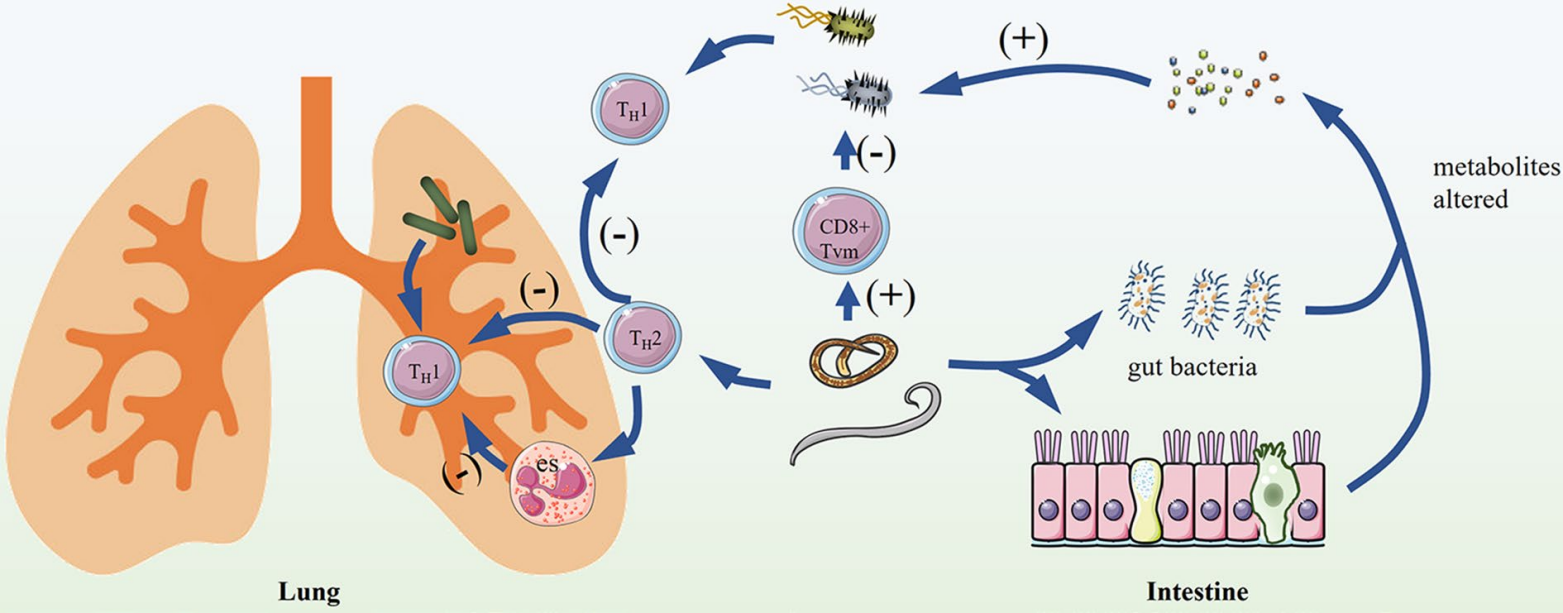
**Co-infection of helminths and bacteria can result in different outcomes**. Improved outcome: Helminth infections can have a protective effect on bacterial infection by increasing the number of virtual memory CD8^+^ T (CD8^+^ TVM) cells. Helminths can improve pulmonary inflammation after bacterial pneumonia through a T_H_2-type immune response associated with eosinophil influx, which limits the development of T_H_1-type immune responses. Worsened outcome: Helminth infection and its ensuing T_H_2 immune response can impair host T_H_1 anti-bacterial response. Helminth infection also causes changes in the small intestinal metabolome from gut microbiota, which contribute to colonization of enteric bacteria.

Apart from these studies, infection of helminths can also have beneficial effects on the outcome of bacterial infections (Figure 1). Infection of *H. polygyrus* protected BALB/c mice that were subsequently infected by *Listeria monocytogenes*. This phenotype was linked to a population of virtual memory CD8^+^ T (CD8^+^ TVM) cells that expanded upon infection with the helminth via IL-4 and IL-4Rα signals [9]. IL-15 and age are also essential for the helminth-induced increase in TVM cells [40, 49]. *H. polygyrus* infection also can enhance acute airway neutrophil responses to *P. aeruginosa* infection to improve survival rates [50]. In addition, prior infection with *Trichinella spiralis* improved pulmonary inflammation and survival after *P. aeruginosa* infection and pneumonia through a T_H_2-type response associated with eosinophils [51]. Helminth infections result in the recruitment of eosinophils that supports persistence and survival by limiting the development of tissue-destructive T_H_1-type immune responses [52].

## Virus and helminth co-infections

**Figure 2:**
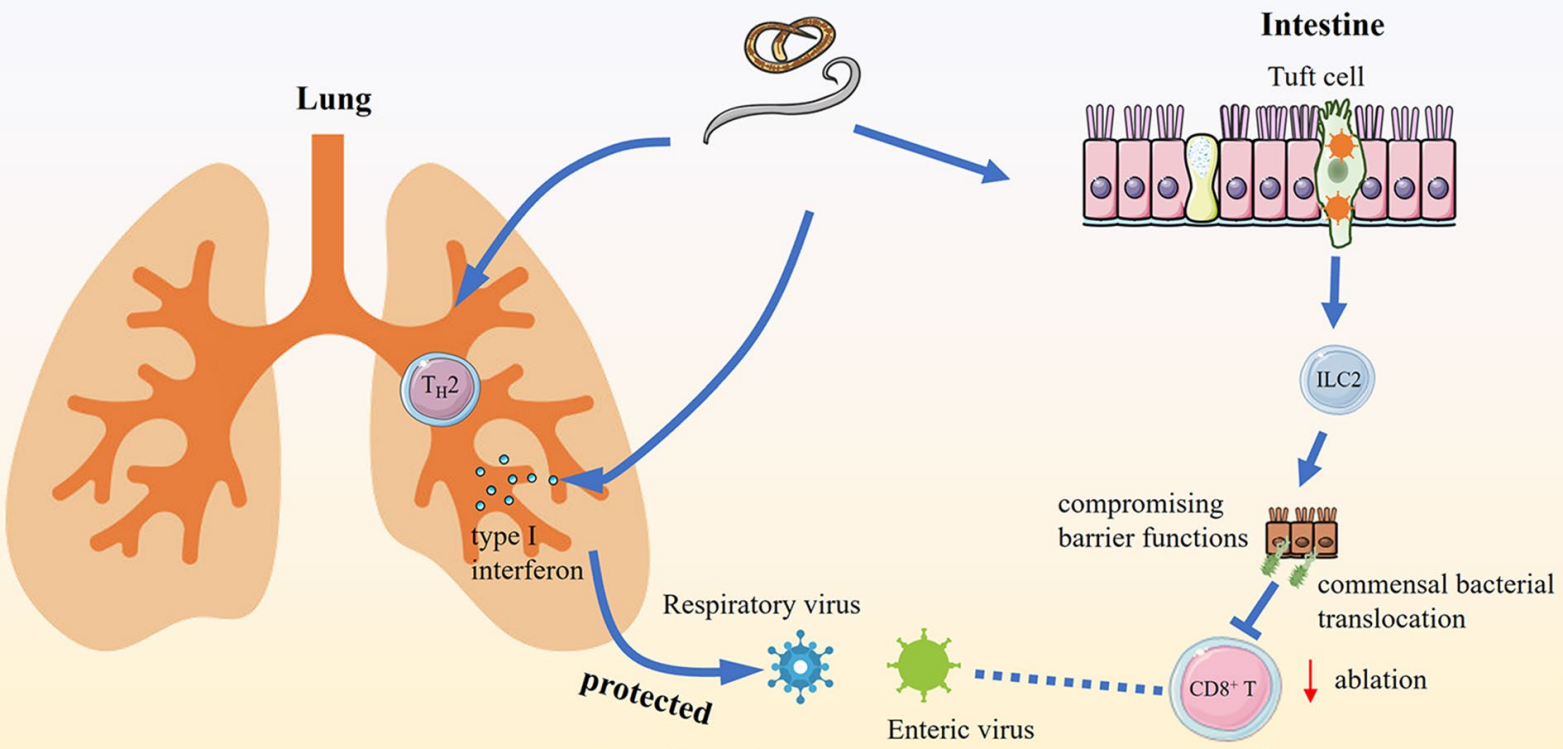
**Co-infection of helminths and viruses can result in different outcomes**. Improved outcome: (*Left*) Enteric helminth infections can improve the outcome of respiratory viral infections via T_H_2 or type I IFN. Worsened outcome: (*Right*) Helminth infections induce type 2 immune response through activation of group 2 innate lymphoid cells (ILC2s) by tuft cells in the intestine. In the setting of some viral infections (e.g., West Nile virus, WNV), enteric helminth infections can lead to impairment of virus-specific CD8^+^ T cells responses through effects on commensal bacterial translocation due to compromised barrier functions. Fewer virus-specific CD8^+^ T cells results in a failure to control systemic infection.

Co-infection of helminths and viruses also can have different outcomes (Figure 2). For example, infection with the helminth *T. spiralis* and the enteric murine norovirus (MNV) resulted in higher viral loads, and this phenotype was dependent on STAT6 signaling and a type 2 cytokine [37]. Co-infection of *H. polygyrus* and WNV exacerbated gastrointestinal tract dysmotility, gut permeability, infection, and mortality via a tuft cell-IL-25-IL-4 receptor signaling axis [33]. In both cases, worm infections stimulated immune cells to secrete IL-4, which polarized macrophages [33, 37] and shifted the immune response from T_H_1 to T_H_2, which impaired control of viral infection in the gastrointestinal tract. As MNV can directly infect and replicate within tuft cells, the type 2 cytokines (IL-4 and IL-25) induced by helminths can promote MNV infection in the setting of tuft cell hyperplasia [53], whereas helminth-dependent effects on WNV pathogenesis required the immunomodulatory functions of tuft cells [33]. Increased viral infection in the lung and greater mortality also were observed in *Ascaris suum* and *vaccinia virus* (VACV) co-infected mice. The ablation of CD8^+^ T cells and the marked reduction of circulating IFN-γ-producing CD4^+^ T cells against VACV were associated with an increase in morbidity and mortality in co-infected animals [54]. Co-infection of *H. polygyrus* or *S. mansoni* eggs reactivated murine gammaherpesvirus (MHV)-68 infection [55]. Treatment with IL-4 complexes plus anti-IFN-γ increased murine γ-herpesvirus infection, suggesting that co-infection can induce reactivation through a “two signal” mechanism. Helminth *N. brasiliensis*-induced type 2 immunity promoted pathology following herpes simplex virus (HSV)-2 infection by an eosinophil influx, which was IL-33/IL-5-dependent but IL-4Rα independent [36]. *H. polygyrus* have been found to exacerbate murine astrovirus infection, as this virus targets goblet cells, which proliferate in response to enteric helminth infection [56]. Suppression of the antiviral type I IFN response by schistosome egg antigens predisposes the liver to enhanced lymphocytic choriomeningitis virus (LCMV, murine pathogen) replication with ensuing immunopathological consequences [57].

In other instances, helminth infection can improve the outcome of viral infections, especially against diseases caused by respiratory viruses. This may be because the host response to infection rather than direct injury of respiratory cells by viral infection accounts for the clinical and pathological changes in the lung [58]. Limiting the infiltration of immune cells to the lungs or changing the quality of their response could lessen pulmonary inflammation [58]. Co-infection with trichinosis reduced influenza virus-induced inflammation in the lungs, although it did not affect viral replication and clearance [38].

During co-infection of *Nematospiroides dubius* and influenza virus in mice, the virus titer in the lungs trended lower than in controls [35]. The progression of H1N1 (A/WSN/33) influenza A virus (IAV) infection can be ameliorated by pre-existing *Litomosoides sigmodontis* infection at larval and juvenile adult stage of filarial infection [59]. *S. mansoni* co-infection affected the pathogenesis of pneumonia virus (PMV) of mice, a mouse virus that models respiratory syncytial virus (RSV) infections. PMV viral burden accumulated more slowly and was cleared by fewer CD8^+^ cytotoxic T cells with less airway inflammation. The increased resistance of coinfected mice to PMV was attributed to TNFα-dependent goblet cell hyperplasia by *S. mansoni* eggs [39]. In a co-infection study of *H. polygyrus* and RSV, enteric helminth infection through pathways that remain undefined induced type I interferon (IFN) signaling in the lung to protect against viral infection, possibly through helminth interactions with the gut microbiota [34]. In a co-infection study with *S. mansoni* and murid gammaherpesvirus 4 (MuHV-4) in mice, helminth infection enhanced control of viral infection by augmenting antigen-specific CD8^+^ T cell responses [40].

Data on helminth co-infection and COVID-19 are just beginning to emerge. Epidemiological studies in Africa suggest that a lower percentage of patients infected with SARS-CoV-2 suffers from serious COVID-19 than in industrialized nations. In some reports, co-infection with *Entamoeba spp*., *Hymenolepis nana*, *S. mansoni*, and *Trichuris trichiura* appeared to lower the probability of developing severe COVID-19 [60]. Consistent with this observation, helminth antigens modulate the activation of CD4^+^ and CD8^+^ T cells of convalescent COVID-19 patients in vitro. Stimulation of peripheral blood mononuclear cells from COVID-19 patients with helminth antigens was associated with increased IL-10 and reduced IFN-γ and TNF-α production [61]. SARS-CoV-2 can trigger over-exuberant immune responses that result in high levels of circulating pro-inflammatory cytokines, which can cause acute respiratory distress and systemic inflammatory response syndromes [62]. As helminths can potently activate anti-inflammatory T_H_2 immune response, this could be a mechanism to mitigate circulatory compromise and lung injury. Beyond this, helminth infection reportedly decreases expression levels of ACE2 receptors, which could lead to reduced SARS-CoV-2 infection in the host [63].

## Conclusions

Co-infection of worms with bacteria or viruses can result in different physiological outcomes, which vary depending on the specific combination of helminth and bacteria or viruses and the niche they occupy. Helminths typically establish chronic infection, and are generally tolerated by the host with limited immunopathology, presumably due to their potent immunomodulatory activity [10]. As a negative consequence of immunomodulation, helminth-infected individuals may be more susceptible to secondary microbial infections, especially when they share a niche [11]. Helminth infections characteristically induce a robust T_H_2 immune response, which can impair the induction of protective T_H_1 immunity against bacterial or viral pathogens [64].

Enteric or cutaneous helminth infection appears to reduce the severity of bacterial or viral infections in the respiratory tract [35, 38, 39, 51, 65], and preliminary results suggest a negative correlation between helminth infection and COVID-19 severity in helminth-endemic regions [66]. During chronic infection, helminths can suppress immune responses to bystander pathogens/antigens and atopic, autoimmune, and metabolic disorders. Helminth-induced immunoregulation occurs through the induction of T_H_2-type cells [66], which can activate macrophage subpopulations that are less inflammatory [67]. Helminths affect subsequent bacterial or viral infections by activating IL-4 signaling pathways [33, 34, 45], including in myeloid cells resulting in their altered transcriptional profile and upregulation of proteins (arginase-1 (Arg-1), chitinase-3-like protein 3, Resistin-like molecule (Relm) α and CD206 (mannose receptor) that decrease inflammatory responses and promote wound healing [68, 69]. The findings of such studies will be very important as they indicate that helminth therapy is a boon for inflammatory diseases.

The hygiene hypothesis purports that in the context of improved hygiene and sanitation, a variety of inflammatory disorders that preferentially affect people in the developed world are linked to a loss of helminth infection. These include asthma, autoimmune diseases (type I diabetes, multiple sclerosis), and inflammatory bowel disease [30]. Helminth infections may alleviate autoimmune diseases by causing a reduction in pro-inflammatory cytokines and immune responses [70]. Helminths that colonize a different niche from where secondary infection with bacteria or viruses occurs also appear to have protective effects by reducing inflammation and immunopathology [3]. Although in many parts of the world, the elimination of helminth infection was considered a success, the emerging awareness of immunological benefits in several different disease contexts warrants reconsideration and even possible targeted re-introduction. The benefits of helminth against infection-related inflammatory diseases such as COVID-19 are very important, especially in the current situation after the COVID-19 pandemic.

During helminth infection, a state of host resistance and tolerance develops, which can impact the course of co-infection by bacteria and viruses. More studies are needed to elucidate the detailed mechanisms of the interactive immune responses that have detrimental effects, as such knowledge could inform future targeted control strategies to avoid the negative outcomes of helminth co-infection. As helminths can also provide benefits to their hosts by virtue of the induced immunoregulatory networks that resolve inflammation and promote wound healing, studies that identify these anti-inflammatory molecules and pathways could be a new source of agents to mitigate adverse pathological inflammation associated with infection or autoimmunity.
